# Advance care planning in dementia care: Wants, beliefs, and insight

**DOI:** 10.1177/09697330211035498

**Published:** 2022-02-10

**Authors:** Annika Tetrault, Maj-Helen Nyback, Heli Vaartio-Rajalin, Lisbeth Fagerström

**Affiliations:** Åbo Akademi University, Finland; Novia University of Applied Sciences, Finland; Åbo Akademi University, Finland; Åbo Akademi University, Finland; University of South-Eastern Norway, Norway

**Keywords:** Advance care planning, autonomy, dementia, relationship-centered care, the qualitative analysis guide of Leuven

## Abstract

**Background:**

Advance care planning gives patients and their family members the possibility to consider and make decisions regarding future care and medical procedures.

**Aim:**

To explore the view of people in the early stage of dementia on planning for future care.

**Research design:**

The study is a qualitative interview study with a semistructured interview guide. The data were analyzed according to the Qualitative Analysis Guide of Leuven.

**Participants and research context:**

Dementia nurses assisted in the recruiting of people with dementia for participation in the study. Study information was mailed to 95 people with early stage dementia. Ten people with dementia and eight caregiver spouses participated in the study.

**Ethical considerations:**

People with dementia belong to a vulnerable patient group, and care was taken in the areas of informed consent and accessible information.

**Findings:**

The views of people with dementia are characterized by a complex storyline involving tensions and movement within the themes of wants, beliefs, and levels of insight. Participants wanted to think about the future but also wanted to live in the here and now.

**Discussion:**

High demands are placed on the advance care planning process for people with dementia and their family caregivers. A dignity-enhancing approach in dementia care emphasizes the dignity of and respect for this vulnerable and care-dependent patient group.

**Conclusion:**

The process of advance care planning in dementia care needs to go beyond person-centered care to a relationship-centered process. The illness trajectory and the impact on autonomy need to be taken into consideration.

## Introduction

Supporting autonomy and engagement in care are at the core of ethical values in healthcare^1^ and important concepts in person-centered care. The person is to be treated as a unique individual and his or her preferences to be taken into consideration. The recommended treatments, risks and benefits, available alternatives, and likely outcomes of no treatment need to be known to the patient.^2^ Life-prolonging care at the end of life is not always in harmony with the needs and intents of the patient.^3,4^ An important way to alleviate this discrepancy is to engage the patient in care decisions,^4,5^ including end-of-life care decisions.

Advance care planning (ACP) is a means of extending patient autonomy to a phase of life where patients are no longer capable of making their own care decisions.^4,6^ It can be described as a process of discussion about the goals for care^4^ and gives patients and their family members the possibility to consider the kind of care and medical procedures that are acceptable or not acceptable in the future.^4,6^ Person-centered care is also a process in which the patient’s life experiences, wishes, and interests are sought as a basis for a care plan^7,8^ and the inclusion of family members is part of the process.^9^ Within dementia care, the challenges of the ACP process reach another dimension as the person with dementia (PWD) will gradually lose cognitive and functional abilities.^2,10–12^ Capability for autonomy should not be seen as the capability to make rational decisions.^13^ Ethical challenges arise when trying to balance a need of PWD for independence and autonomy with the degree to which lost abilities affect decision-making capacity.^14^

This study is part of a project which aims to develop a model for a relationship-centered ACP process in dementia care. The objective of the current study is to explore the view of people in the early stage of dementia on planning for future care. How do they describe and understand their current circumstances and their ability to affect their future situation?

## Background

Dementia is a global issue of concern with the number of people living with dementia expected to double every two decades.^15^ In recent years, ACP in dementia care has received attention with a number of studies exploring barriers and facilitators as well as subsequent effects on end-of-life care.^6,16,17^ A challenge in the ACP process is the need for guidance pertaining to the timing of ACP as well as to the approach chosen when introducing the ACP concept to patients and their family members.^10^ A survey of general practitioners’ perceptions on ACP for people with dementia (PWDs) indicated that most respondents agreed that discussions in the early stages would make decision-making easier during the advanced stage of the disease. However, many were reluctant to holding these discussions at the time of diagnosis. The optimal timing was viewed as being determined by the readiness of the patient and family to acknowledge the end-of-life considerations.^18^

Many ACP programs and interventions have been developed as evidenced by the results of a recent scoping review.^19^ There is a general lack of dementia-specific components in a number of the identified ACP programs and interventions. A low number of published ACP intervention studies include feedback on the interventions from the people with dementia themselves.^19^

In Finland, palliative care has received attention during the last two decades with reports and recommendations produced by the Ministry of Social Affairs and Health.^20^ Concepts related to ACP, such as advanced directives, advance decisions to refuse treatment, and lasting power of attorney, have been in use for a long time.^21^ However, ACP has not received attention in Finland until fairly recently,^20,21^ and thus, there are few relevant studies.^21^

## Methodology

### Research setting

The context is dementia care in the Finnish welfare society. Finland is a developed country with an established home-nursing and long-term care infrastructure according to the “Nordic model,” characterized by strong institutionalized care for older people.^22,23^ In a society with a government-financed healthcare and social care system, the population lives with the beliefs, hopes, and expectations that society will take care of the ill and the older people, and that family members and relatives will not be expected to provide and finance long-term care.^24^

### Design

The study is a qualitative interview study with an inductive approach and a semistructured interview guide (Supplemental file 1). The interview guide was constructed by two of the research team members and evaluated and approved by the remaining two team members. The general area of inquiry revolved around the wishes of the PWD since being diagnosed with dementia and views on planning for future care in general. The interview guide consisted of open-ended key questions with optional and flexible follow-up questions to elicit descriptive answers to the main inquiry. Care was taken to use language that the participants could easily understand. The dementia nurses from memory clinics in four municipalities were asked to assist in the recruiting of PWDs, as they know their clients well. Before recruitment started, one dementia nurse was asked to evaluate the recruitment form.

### Inclusion criteria

Study information and recruitment forms were mailed to 95 people in four municipalities. The recipients were all living in their own homes as opposed to assisted living facilities. None of the participants had regular home care services at the time of the study. Due to confidentiality reasons, the research team did not have access to the client registers. The dementia nurse determined which of her clients would receive the study information and recruitment form. The decision was based on the nurses’ own assessment of the cognitive abilities and illness insight of the PWD, as understanding of the purpose of the interview and the ability to give informed consent were necessary. The dementia nurses were asked to keep careful records of the number of recruitment forms mailed out as well as the age span of the recipients. A certain minimum number of points achieved in Mini Mental State Examination or Consortium to Establish a Registry for Alzheimer’s Disease testing were not set as a criterion as there is disagreement about whether these fully indicate and assess the capabilities of the PWD to understand and participate in an interview situation.^25–27^ A limit was not set on time since diagnosis as the progress of dementia is highly individual and time since diagnosis is not an indicator of ability to participate in a study.^28^

### Participants

Ten people, aged 65–85 (mean age 76.6 years), accepted the invitation to participate and were equally distributed in gender. The interviews took place in participants’ homes. The researcher was contacted by three PWDs themselves and by caregiver spouses on behalf of the remaining PWDs. At first contact with the spouse, it was emphasized that the PWD him/herself had to be able to give informed consent. Two of the participants and their caregivers were not sure of which type of dementia they had been diagnosed with. The characteristics of the participants are presented in Table 1.

**Table 1.** Participants’ information.

Participant	Gender	Age, years	Time since diagnosis	Diagnosis	CG present	CG gender	Age, years
**1**	Female	85	1 year	Alzheimer’s	X	Male	89
**2**	Male	79	6 months	Unknown	X	Female	71
**3**	Male	82	3.5 years	Alzheimer’s	X	Female	79
**4**	Male	71	3 years	Alzheimer’s	X	Female	67
**5**	Female	76	1 month	Unknown		–	–
**6**	Male	82	2 years	Alzheimer’s	X	Female	80
**7**	Female	83	5 months	Alzheimer’s		–	–
**8**	Female	75	1 year	Alzheimer’s	X	Male	81
**9**	Female	65	3 months	Alzheimer’s	X	Male	69
**10**	Male	68	4 years	Benson’s syndrome	X	Female	65

CG: caregiver spouse.

During eight of the interviews, the PWD was accompanied by his or her caregiver spouse as per the wishes of the PWDs and the spouses, making the interviews dyadic in nature. The caregiver spouses sometimes expressed their own views on ACP and supported the PWDs by at times clarifying statements. However, precedence was given to the PWD responses as the primary source of information. Two PWDs were interviewed without a caregiver spouse as they were both widowed. Data were collected from July 2018 to April 2019. The interviews lasted between 28 and 85 min with the average interview lasting about 60 min. The interviews were recorded with the permission of the PWD and the caregiver spouse (when present), and transcribed verbatim. Immediately after each interview, the interviewer recorded field notes, observations, and reflections on the interview. Field notes were also transcribed verbatim.

### Data analysis

The current study used a modified version of the Qualitative Analysis Guide of Leuven (QUAGOL).^29,30^

**Figure 1.**
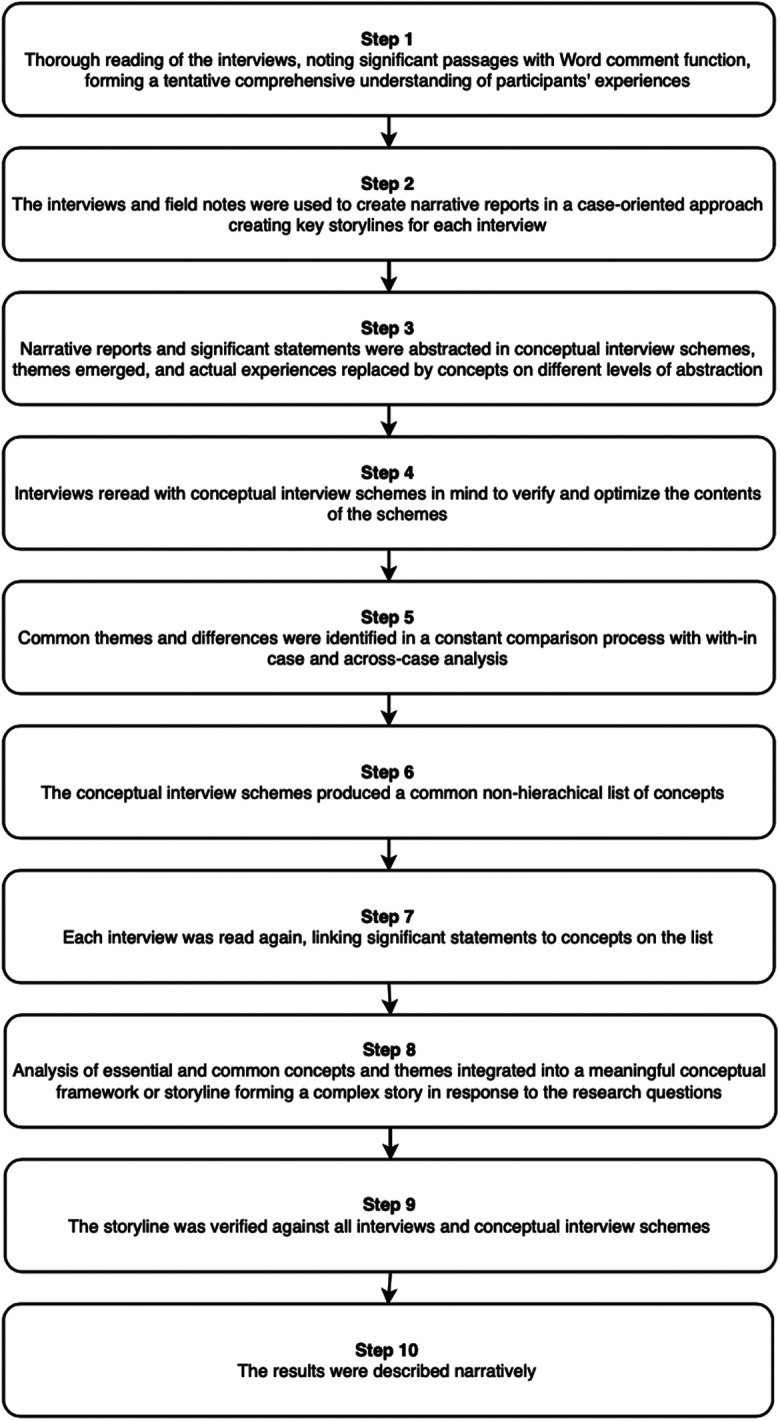
Stages of the analysis.

QUAGOL presents a multifaceted, comprehensive, and systematic approach to the analysis of complex qualitative data without being rigid. A systematic analytical approach is combined with a case-oriented narrative approach.^30^ The analysis was performed in interconnected stages described in Figure 1. The analysis process was a team activity where forthcoming results were discussed continuously within the research team. Common themes and differences between emergent findings were explored. An example of a conceptual interview scheme can be found in Supplemental file 2.

### Ethical considerations

This study is ethically challenging as PWDs belong to a vulnerable patient group where heightened sensitivity is required. The researcher needs good understanding of guidelines with regards to informed consent, the balance of risk and benefits, and insight into acceptable procedures for such patient groups.^31^ An approved application for permission to conduct the study was obtained from the ethics committee of Åbo Akademi University and from the healthcare committee of each municipality participating in the study. Participants were recruited specifically among people in the early stage of dementia that the dementia nurse deemed capable of understanding the purpose of the study and participating. The researchers consciously used plain language in the consent form and the interviewer reviewed the consent form with each participant and their caregiver spouse before the interview. Participants were informed of their right to withdraw at any time, interview confidentiality, and how their identity would be protected. They were told about the potential discomfort experienced when discussing illness progression and end-of-life care. At the end of the interview, the participants were told they could contact the researcher with further questions or concerns. They were also reminded of the local memory clinic and dementia nurse as support systems.

## Results

The results are presented in the form of a narrative storyline. The participants gave an account of their views on ACP as well as of their fears and hopes for the future. The views of the PWDs and their caregiver spouses are characterized by a complex dialectic tension between and within the three main themes of the conceptual framework created in the analysis: wants, beliefs, and levels of insight. Within this storyline, there is tension and movement between the poles.

### Wants: to plan for the future or to live for today

Participants fell mainly into two groups, one that thought ahead by anticipating future needs and possible consequences of illness progression and one that did not want to think about or be reminded of what the future might hold. This latter group wanted to live day by day and not worry too much about the future. Some participants vacillated between the viewpoints by understanding the need to think about the future but making a conscious decision to live in the present and not worry about the future. Most participants felt positively about ACP in general while expressing preference for living day by day. Some expressed the view that since their illness was progressing slowly, they did not feel an urgent need to plan for or think about the future. A few PWDs and caregiver spouses noted the importance of planning sooner rather than later and were willing to accept help with planning for the future.

PWD3: *No, one doesn’t want to dwell on it, how things may become and so on.*

Two of the PWDs disclosed other health conditions which they felt would lead to death before the dementia would. They expressed the wish that they would indeed die from the other illness before the dementia worsened. Participants did not want life-prolonging care if there were no hope for improvement.

PWD4: *I haven’t been able to decide how I would want this bu-but for sure it’s the way that…if the cancer doesn’t progress fast enough, it means that I will get more and more memory problems…and-and that’s something that I don’t want. My memory would become really bad that way…so in that sense you could say that I actually would rather die from cancer…and as quickly as possible then, if it…then when it becomes difficult.*

Almost all of the participants had completed legal documents such as continuing power of attorney where the adult children were assignees. In all of the PWD–caregiver spouse dyads, the spouse had taken over management of household finances as well as contact with healthcare organizations. In three of the dyads, a wish to avoid family conflicts caused by inheritance issues was mentioned as a motivating factor in drawing up legal documents. Few of the participants had completed living wills or advance directives. One of the dyads had obtained living will forms but found them too complicated to complete and wished for a simpler document to be made available. Some expressed the view that it was enough to have talked to their spouse and/or children about end-of-life care and stated that as a reason for not having completed a living will. They trusted their spouse or children to make good choices for them. When asked about how they would feel about a nurse visiting them in their homes to inform them about living wills, most participants were positive to the suggestion while a few said they were not interested in such an arrangement.

PWD4: *I do want that on paper, in the way that one shouldn’t be tortured until the end, always to the end in that way.*

Participants who took the view that they wanted to live for today expressed a desire to not burden themselves and their spouses with worry about the future. They wanted to enjoy the present moments and not feel stressed about the future. The view that anything might happen in the future was expressed, and thus planning for it is pointless. Others again expressed a desire to feel some sort of control over the future.

CG10: *No, we haven’t thought about it. Someone gets to, someone gets to decide that later. No, that [end-of-life] is so, it’s so dismal somehow since we’ve always felt ourselves to be so youthful and thought that we have so much left…somehow one wants to sweep it under the rug and just enjoy the sun today and simply not care about it.*

Half of the PWD–caregiver spouse dyads had simplified their living arrangements by moving from single-family homes to easier to care for apartments. The widowed participants felt stressed about the future in anticipation of worsening illness. Both had made plans for future living arrangements in nearby assisted living facilities.

### Beliefs: to be cared for with love or to be a burden

The PWDs were in most cases aware of the difficulties they would have if their spouse would not be there to help them. They expressed their gratitude while also seeming to take for granted that the spouse would be there for them. One PWD did not even want to think about the possibility that the caregiver spouse would be the first to die, thereby leaving the PWD behind.

PWD4: *I don’t know…no, I can’t really imagine that-that it would be that way that you wouldn’t be…I don’t know what I’d do if something were to happen so that you were not here, I don’t know. I haven’t thought about it that way. For sure I don’t want to think about it…consciously at least.*

A few acknowledged that they would feel like a burden for their caregiver spouse if or when their condition worsened and expressed a wish to move to a nursing home in such circumstances. Some of the dyads had talked to each other about end-of-life care while others had not. In several instances, the couples assumed the other would know without needing to discuss it. The participants had experienced the creation of the welfare state during their lifetime. The participants trusted healthcare in general and did believe that there will be enough nursing home places for everyone as needed.

CG3: *We kind of trust society, we have tried to be a part of building our society…we trust our society…that there will be people who want it to function.*

PWD8: *[when I become really ill] it’ll be the bed ward then and I’ll lay there until I get to go to some other place.*

Most of the participants had personal experiences of their loved ones or friends becoming ill and dying from dementia. The experience influenced some of the participants, particularly in the way they viewed nursing homes and where they wanted to live and be cared for in the future. The ones who had visited loved ones in nursing homes had strong opinions about what a good nursing home and care received is like. Recent national nursing home scandals did, however, affect the participants. They expressed doubts and fears that there may not be nursing home vacancies when needed and that the care received would not be of high quality.

### Insight: to be aware of progressing illness or to think things will stay the same

While some participants had educated themselves about dementia, others were less knowledgeable about the illness. Most of the PWDs seemed to have trouble imagining a future where their condition had worsened. Several of the PWDs used the phrase “IF it gets worse” rather than “WHEN it gets worse.” Some of the caregiver spouses also seemed to have difficulties imagining a worsening condition. The slowness of the illness progression was mentioned as a factor in this. The caregiver spouses also tended to use the term “if” instead of “when.” Two of the PWDs had professionally cared for patients with dementia in nursing homes or mental institutions, but did not seem to connect those experiences to their own illness and possible illness trajectory.

Interviewer: *What do you think the future could be like?*

PWD8: *Exactly the way it is now*

CG8: *Well, no*

PWD8: *if we both live*

CG8: *yes, but it can*

PWD8: *(raising her voice) yes but I said for as long as we both are alive and I’m healthy then it will be like it is today but we don’t know anything about it when that day comes, it can come tomorrow*

Some PWDs were reluctant to acknowledge any difficulties or lost functions, while others were very much aware of losses. One identified irritation as well as increased anxiety associated with being confronted with unfamiliar things. One of the widowed participants worried about the illness progressing and not knowing the speed of progression.

PWD7: *…but I don’t know how it will progress and how fast it will go and that’s why I worry so much about why it’s taking such a long time [to get an appointment for a follow-up],…I know that I have a terrible time with collecting myself and to discuss and I forget all kinds of things, not just names but…words…that’s the worst, not knowing how long I can be at home for, when will I have to go there [nursing home] and I don’t want to go there…*

In two of the care dyads, the relationships were complicated by the PWD’s lack of insight into the help they needed and how much effort was made by the spouse. The PWD even bristled at times at the suggestion that they would not be able to, for example, live at home on their own or partake in travel on their own.

None of the participants felt that it was a negative or burdening experience to talk about ACP and end-of-life care. Some of the caregiver spouses expressed gratitude for the opportunity to talk about their situation and for the attention they felt dementia patients were given throughout this study.

## Discussion

Dementia care is a complicated process, with policies and care plans often guided by our ethical values. While participants in the current study acknowledged a need to plan for the future, they also expressed a wish to live in the present and not bother themselves too much with gloomy thoughts. While thinking about the future elicited worries and fears, there was simultaneously hope that the illness would somehow not progress. There were also thoughts about other illnesses leading to death before the dementia worsened, thereby eliminating the need to plan for future care. PWDs wanted and trusted their spouse to care for them, but did not want to be or become a burden. They believed and trusted that society would take care of them if needed while harboring doubts about the quality of older people care in general and future access to a place in a nursing home. Most of the PWDs were aware of lost functions, but were at times not aware of how the illness affected them and their caregiver spouse. Such powerful dialectic tensions between and within wants, beliefs, and insight place high demands on the ACP process for PWDs and their family caregivers.

Timing is of importance in ACP discussions and even more so in dementia care as the PWD will progressively lose cognitive and functional abilities.^10–12^ Time is required to come to terms with the diagnosis, but is also important when discussing future care decisions in the early stages of the disease.^32,33^ A few of the PWDs showed signs of anosognosia, lacking insight into their illness. All seemed to have difficulties imagining a future where the illness had progressed and what it would mean for themselves and their caregiver spouse. Similar results have been found in previous studies with PWDs.^16,34,35^ Most study participants did not seem to recognize dementia as a life-limiting illness. Some seemed to view dementia as a normal part of aging, especially when reflecting over the illness trajectories of their own parents or older relatives with dementia. These barriers to ACP have been identified in other studies as well.^36,37^

To enable PWDs to make informed decisions about care, they need information about the possible illness trajectory, different care alternatives, and the consequences of these alternatives. Informative discussions can take place as part of a systematic, person-centered ACP process in the early stage of dementia while the PWD has the necessary capacity to partake in decision-making about future care. The term “person-centered” care was first used by Kitwood in the context of dementia care,^38,39^ emphasizing the lived experience of PWDs along with the importance of communication and relationships.^38,40^ This carries over to Gastmans’s^41,42^ foundational ethical framework for dignity-enhancing nursing care, where lived experience and the dialogical-interpretative process are two of the framework’s pillars. Gastmans argues for a move from principalism where respect for an autonomy that is cognitive-oriented is one of the cornerstones. Dignity-enhancing care for PWDs offers an alternative, with an emphasis on the respect for and dignity of vulnerable and care-dependent people in their full reality. Care practices have to be situated in a relational and dialogical context.^42^ When this frame of mind is brought to the ACP process in dementia care, it opens up for a dialogical-interpretative process based on lived experience with the aim of protecting and maintaining the dignity of the PWD. The ethical challenges in dementia care move to the forefront when it comes to illness trajectory—do PWDs want information about illness progression and about what can be expected in the late stages? Such information can aid in decision-making about future care, but also promote a sense of hopelessness and despair.

The place of care for PWD transitions from out-patient memory clinics to assisted living facilities and nursing homes as the dementia progresses and the PWD’s care needs increase. A challenge in dementia care is this lack of continuity in contact with care professionals. In this type of dementia care structure, the PWD and his or her family caregiver will encounter many nurses and doctors along the illness trajectory who are not necessarily familiar with the PWD and his or her family, their care preferences, and their wishes for end-of-life care. A focus on the restructuring of dementia care to ensure care continuity, staff competency, and responsibility is essential. Knowing the patient^43^ becomes of utmost importance in the dementia care ACP process when striving to maintain dignity and protect this vulnerable patient group. A relationship-centered dementia care model where the nurse works together with the PWD and the family caregiver in a triad, to promote senses of continuity, security, purpose, achievement, and significance is ideal.^44,45^

PWDs account for a large minority group within all populations worldwide and need to be treated as citizens with personhood^46^; however, persons with diminishing cognition were rarely heard from in research despite the growth in size of this group.^47,48^ In the late 1990s and early 2000s, with the increased interest in person-centered care came a growing recognition in the research community that PWDs should be included in the research as participants and not merely as subjects or objects. It is possible to include PWDs in research and it is important to do so.^49^

### Strengths and limitations

There are several limitations in this study. One of the challenges in involving PWDs in research pertains to reaching a wider range of participants, such as people who do not have family caregivers and people who lack illness insight. The sample is limited in size and does not reflect the full range of PWDs, as the recruitment of enough participants for the study was challenging. Therefore, the findings cannot be generalized to the study population. It should additionally be noted that the findings were potentially biased. The people who contacted the researcher were aware of their diagnosis and possibly already interested in planning for future care. Furthermore, the presence and participation of the caregiver spouse and how this might have affected the responses needs to be noted as well.^50^ The interviews conducted jointly resulted in a shared narrative,^51^ where there is the risk that the voice of the PWDs is overpowered.^52^ Other disadvantages may include the interviewer only getting the “public” story.^50^ However, dyadic interviewing is also considered a method of triangulation and as an accommodation for PWDs.^50^

QUAGOL^29,30^ was used as the guiding tool it is intended for. The potential stumbling blocks of the method include information overload, losing track of the research question, and the focus on intuition and creativity.^29,30^ The trustworthiness of the analysis process was enhanced by the documentation of reflections and field notes which were used in the narrative reports and conceptual interview schemes. Continuous research team discussion about data analysis and emergent results affirm credibility.

## Conclusion

The process of ACP in dementia care needs to extend beyond person-centered care to a relationship-centered process, and it needs to consider the illness trajectory as well and the impact of the illness on autonomy. To take willingness as well as reluctance to plan for the future into account is possible for a nurse who knows the patient. Promoting the sense that society can be trusted to care for PWDs is essential for the well-being of PWDs and perhaps even more so for family caregivers.

## Supplemental Material

sj-pdf-1-nej-10.1177_09697330211035498 – Supplemental Material for Advance care planning in dementia care: Wants, beliefs, and insight Click here for additional data file. Supplemental Material, sj-pdf-1-nej-10.1177_09697330211035498 for Advance care planning in dementia care: Wants, beliefs, and insight by Annika Tetrault, Maj-Helen Nyback, Heli Vaartio-Rajalin and Lisbeth Fagerström in Nursing Ethics

sj-pdf-2-nej-10.1177_09697330211035498 – Supplemental Material for Advance care planning in dementia care: Wants, beliefs, and insight Click here for additional data file. Supplemental Material, sj-pdf-2-nej-10.1177_09697330211035498 for Advance care planning in dementia care: Wants, beliefs, and insight by Annika Tetrault, Maj-Helen Nyback, Heli Vaartio-Rajalin and Lisbeth Fagerström in Nursing Ethics

## References

[1] World Health Organization Europe. Better palliative care for older people. http://www.euro.who.int/__data/assets/pdf_file/0009/98235/E82933.pdf (2004, accessed 12 April 2021).

[2] Brummel-Smith K Halperin S . Patient-centered care for people with cognitive impairment is possible in primary care. J Am Soc Aging 2013; 37: 87–91.

[3] Teno JM Fisher ES Hamel MB , et al. Medical care inconsistent with patients’ treatment goals: association with 1-year Medicare resource use and survival. J Am Geriatr Soc 2002; 50(3): 496–500. 11943046 10.1046/j.1532-5415.2002.50116.x

[4] Russell S Detering K . What are the benefits of advance care planning and how do we know? In: Thomas K Lobo B Detering K (eds) Advance care planning in end of life care. 2nd ed. Oxford: Oxford University Press, 2017, pp. 17–25.

[5] Rietjens J Korfage I van der Heide A . Advance care planning: not a panacea. Palliat Med 2016; 30(5): 421–422. 27095742 10.1177/0269216316642963

[6] Rietjens JAC Sudore RL Connolly M , et al. Definition and recommendations for advance care planning: an international consensus supported by the European Association for Palliative Care. Lancet Oncol 2017; 18(9): e543–e551. 28884703 10.1016/S1470-2045(17)30582-X

[7] Kitwood T . Toward a theory of dementia care: ethics and interaction. J Clin Ethics 1998; 9(1): 23–34. 9598430

[8] Edvardsson D Backman A Bergland Å , et al. The Umeå ageing and health research programme (U-Age): exploring person-centred care and health-promoting living conditions for an ageing population. Nord J Nur Res 2016; 36(3): 168–174.

[9] Ewing G Austin L Diffin J , et al. Developing a person-centred approach to care assessment and support. Br J Community Nurs 2015; 20(12): 580–584. 26636891 10.12968/bjcn.2015.20.12.580

[10] Cotter VT Spriggs M Razzak R . Advance care planning in early stage dementia. J Nurse Pract 2018; 14(3): 142–147.

[11] Dening K Jones L Sampson E . Advance Care Planning for people with dementia: a review. Int Psychogeriatr 2011; 23: 1535–1551. 21867597 10.1017/S1041610211001608

[12] van der Steen JT van Soest-Poortvliet MC Hallie-Heierman M , et al. Factors associated with initiation of advance care planning in dementia: a systematic review. J Alzheimers Dis 2014; 40(3): 743–757. 24531163 10.3233/JAD-131967

[13] Nuffield Council on Bioethics. Dementia: ethical issues. London: Nuffield Council on Bioethics, 2009.

[14] Dening K Sampson EL De Vries K . Advance care planning in dementia: recommendations for healthcare professionals. Palliat Care 2019; 12: 1178224219826579.

[15] Livingston G Sommerlad A Orgeta V , et al. Dementia prevention, intervention, and care: 2020 report of the Lancet Commission. Lancet 2020; 396: 413–446. 32738937 10.1016/S0140-6736(20)30367-6 PMC7392084

[16] Dickinson C Bamford C Exley C , et al. Planning for tomorrow whilst living for today: the views of people with dementia and their families on advance care planning. Int Psychogeriatr 2013; 25(12): 2011–2021. 24053783 10.1017/S1041610213001531

[17] Hirschman KB Kapo JM Karlawish JH . Identifying the factors that facilitate or hinder advance planning by persons with dementia. Alzheimer Dis Assoc Disord 2008; 22(3): 293–298. 18580595 10.1097/WAD.0b013e318169d669 PMC2666935

[18] Brazil K Carter G Galway K , et al. General Practitioners Perceptions on advance care planning for patients living with dementia. BMC Pall Care 2015; 14: 14.

[19] Tetrault A Nyback M-H Vaartio-Rajalin H , et al. Advance Care Planning interventions for older people with early-stage dementia: a scoping review. Nord J Nurs Res. Epub ahead of print 20 May. DOI: 10.1177/20571585211014005.

[20] Saarto T and Expert Working Group. Providing palliative treatment and end-of-life care. Helsinki: Ministry of Social Affairs and Health, 2017, p. 44.

[21] Lehto J Marjamäki E Saarto T . Elämän loppuvaiheen ennakoiva hoitosuunnitelma [Advance care planning with early plan for end-of-life care]. Lääketieteellinen aikakausikirja Duodecim 2019; 135(4): 335–342. https://www.duodecimlehti.fi/duo14788

[22] Sapir A . Globalisation and the reform of European social models. J Common Mark Stud 2006; 44(2): 369–390.

[23] Daly M Lewis J . The concept of social care and the analysis of contemporary welfare states. Br J Sociol 2000; 51(2): 281–298. 10905001 10.1111/j.1468-4446.2000.00281.x

[24] Fernández-Alonso M Jaime-Castillo AM . Welfare state and individual expectations of economic support: a comparison of Norway and Spain. Int Sociol 2016; 31(1): 37–56.

[25] Bassett SS . Attention: neuropsychological predictor of competency in Alzheimer’s disease. J Geriatr Psychiatry Neurol 1999; 12(4): 200–205. 10616868 10.1177/089198879901200406

[26] Kim SYH Caine ED . Utility and limits of the mini-mental state examination in evaluating consent capacity in Alzheimer’s disease. Psychiatr Serv 2002; 53(10): 1322–1324. 12364686 10.1176/appi.ps.53.10.1322

[27] Gregory R Roked F Jones L , et al. Is the degree of cognitive impairment in patients with Alzheimer’s disease related to their capacity to appoint an enduring power of attorney. Age Ageing 2007; 36(5): 527–531. 17913758 10.1093/ageing/afm104

[28] Stages of Alzheimer’s. https://www.alz.org/alzheimers-dementia/stages (accessed 12 April 2021).

[29] Dierckx de Casterlé B Gastmans C Bryon E , et al. QUAGOL: a guide for qualitative data analysis. Int J Nurs Stud 2012; 49(3): 360–371. 21996649 10.1016/j.ijnurstu.2011.09.012

[30] Dierckx de Casterlé B De Vliegher K Gastmans C , et al. Complex qualitative data analysis: lessons learned from the experiences with the qualitative analysis guide of Leuven. Qual Health Res 2020; 31: 1083–1093. 33135554 10.1177/1049732320966981

[31] Polit D Tatano Beck C . Nursing research. Generating and assessing evidence for nursing practice. 9th ed. Philadelphia, PA: Lippincott, Williams & Wilkins, 2012.

[32] Orsulic-Jeras S Whitlatch CJ Szabo SM , et al. The SHARE program for dementia: implementation of an early-stage dyadic care-planning intervention. Dementia 2016; 18: 360–379. 27738110 10.1177/1471301216673455

[33] Poppe M Burleigh S Banerjee S . Qualitative evaluation of advanced care planning in early dementia (ACP-ED). PLoS ONE 2013; 8(4): e60412. 23630571 10.1371/journal.pone.0060412 PMC3629937

[34] de Boer ME Dröes R-M Jonker C , et al. Thoughts on the future: the perspectives of elderly people with early-stage Alzheimer’s disease and the implications for advance care planning. AJOB Prim Res 2012; 3: 1422.

[35] Clare L . Managing threats to self: awareness in early stage Alzheimer’s disease. Soc Sci Med 2003; 57(6): 1017–1029. 12878102 10.1016/s0277-9536(02)00476-8

[36] Dempsey D . Advance care planning for people with dementia: benefits and challenges. Int J Palliat Nurs 2013; 19(5): 227–234. 23971306 10.12968/ijpn.2013.19.5.227

[37] Dening K Jones L Sampson EL . Preferences for end-of-life care: a nominal group study of people with dementia and their family carers. Palliat Med 2013; 27(5): 409–417. 23128905 10.1177/0269216312464094 PMC3652642

[38] Brooker D . Person-centred dementia care: making services better, 2006, http://ebookcentral.proquest.com

[39] Fazio S Douglas P Flinner J , et al. The fundamentals of person-centered care for individuals with dementia. Gerontol 2018; 58: S10–S19.

[40] Kitwood T . The experience of dementia. Aging Ment Health 1997; 1(1): 13–22.

[41] Gastmans C . Dignity-enhancing nursing care: a foundational ethical framework. Nurs Ethics 2013; 20(2): 142–149. 23466947 10.1177/0969733012473772

[42] Gastmans C . Dignity-enhancing care for persons with dementia and its application to advance euthanasia directives. In: Denier Y Gastmans C Vandevelde A (eds) Justice, luck & responsibility in health care. Dordrecht: Springer Science + Business Media, 2013, pp. 145–165.

[43] Fagerström L (ed.). Avansert klinisk sykepleie *[Advanced* practice nurse]. Oslo: Gyldendal, 2019.

[44] Keady J Nolan M . The dynamics of dementia: working together, working separately, or working alone? In: Nolan M Lundh U Grant G , et al. (eds) Partnership in family care. London: Open University Press, 2003, pp. 15–32.

[45] Nolan MR Davies S Brown J , et al. Beyond “person centred” care: a new vision for gerontological nursing. J Clin Nurs 2004; 13(3a): 45–53. 15028039 10.1111/j.1365-2702.2004.00926.x

[46] Dewing J. From ritual to relationship. A person-centred approach to consent in qualitative research with older people who have dementia. Dementia 2002; 1: 157–171.

[47] Skovdahl K Dewing J . Co-creating flourishing research practices through person-centred research: a focus on persons living with dementia. In: McCormack B van Dulmen S Eide H , et al. (eds) Person-centred healthcare research. Hoboken, NJ: Wiley Blackwell, 2017, pp. 85−93.

[48] Bartlett R O’Connor D . Broadening the dementia debate: towards social citizenship. Bristol: The Policy Press, 2010.

[49] Hellström I Nolan M Nordenfelt L , et al. Ethical and methodological issues in interviewing persons with dementia. Nurs Ethics 2007; 14(5): 608–619. 17901172 10.1177/0969733007080206

[50] Bell BL Campbell V . Dyadic interviews in qualitative research *(Research Shorts Series #1)*. Charlottetown, PE: Young Lives Research Lab, University of Prince Edward Island, 2014.

[51] Arskey H . Collecting data through joint interviews. Soc Res Update 1996; 15: 1–8.

[52] Caldwell K . Dyadic interviewing: a technique valuing interdependence in interviews with individuals with intellectual disabilities. Qual Res 2014; 14(4): 488–507.

