# Supplementary material for: Advance care planning in dementia care: Wants, beliefs, and insight
Source: Nurs Ethics. 2022 Feb 10;29(3):696–708. doi: 10.1177/09697330211035498 (PMC9127932; doi:10.1177/09697330211035498)
Supplement: sj-pdf-1-nej-10.1177_09697330211035498 – Supplemental Material for Advance care planning in dementia care: Wants, beliefs, and insight [file sj-pdf-1-nej-10.1177_09697330211035498.pdf]

Conceptual interview scheme - Interview 3

Male, 82 years old, Alzheimer diagnosis 3,5 years ago

Central Question: **How do PWDs describe and understand their current circumstances and their ability to affect their future situation?**

Central experiences

- They feel that they have not received **information and support** with regards to the Alzheimer's diagnosis. They have looked for information on their own
- A slowly progressing illness has eased the anxiety but the **loss of abilities** is still obvious, i.e. biking, running, walking without walking canes, driving, hearing, a second language. The PWD is aware of the losses.
- The **loss of activities**: socializing, club activities, participation in local politics, grocery shopping, reading, TV-watching, computer use, garden tasks. The CG has also down sized club activities.
- The PWD is **motivated to keep active** with physical therapy and lawn mowing.
- The PWD feels **alienated and frustrated** due to bad hearing in combination with difficulties to follow conversations.
- They **do not connect** the physical problems with Alzheimer's.
- The PWD is trying to compensate memory lapses by continuously taking notes.
- There is **insight** that it will get worse. The word ”when” is used and not the word ”if”.
- There is no need for home services but they are **willing to accept help** when needed.
- They feel that **care staff can be trusted**
- The communication between PWD and CG is important and has always worked, there is **contact, respect and trust.**
- They **trust** the other one to make the right decisions as needed.
- They have **not experienced** loved ones suffering from dementia.
- There is insight that every-day life is manageable since they live **together**, it would not work w/o the CG.
- It is important to be able to keep on **living at home**.
- It is important that **future help and support is given in the home** to avoid stress and anxiety for the PWD.
- It is important that there is a nursing home in their own village to make it easier on family members and **to know the care staff**.
- They find it **difficult to know what the future entails**.
- They **do not want to discuss future problems** but they do **want to discuss current problems** in the here and now.
- They **do not want to think about the future**, thinking about the future brings **negativity and depression**.
- They have **never been the types to fret** over future worries, they want to **face the day as it comes,** anything can happen.

Key concepts:

- **The loss of abilities and activities**
- **Insight about progressing illness**
- **Care staff can be trusted**
- **The caregiver spouse can be trusted**
- **Don't want to think about the future**
