# Supplementary material for: Advance care planning in dementia care: Wants, beliefs, and insight
Source: Nurs Ethics. 2022 Feb 10;29(3):696–708. doi: 10.1177/09697330211035498 (PMC9127932; doi:10.1177/09697330211035498)
Supplement: sj-pdf-2-nej-10.1177_09697330211035498 – Supplemental Material for Advance care planning in dementia care: Wants, beliefs, and insight [file sj-pdf-2-nej-10.1177_09697330211035498.pdf]

Interview guide

Can you tell me about the information you have received about your illness?

- Did you understande the information? Is there something you would like to know more about?
- What was your experience of the care received in connection with the investigation and when you received your diagnosis?

What is your opinion on planning for future care already in this early stage of illness? Have you thought about how you would want to be cared for at the end stage of the illness?

- Have you talked to anyone about it?
- Do you want to talk to care staff or your doctor in advance?
- Do you have experience of dementia in close relatives or friends? How have you been affected by that experience?

Who do you want to make decisions for you when you are no longer able to make your own decisions?

- Does that person know your wishes?
- Which role do you want your family to have in decision making?

What is your view on living wills?

- Would you want your wishes to be documented in a living will? Why? Why not?

Would you like to add something?
How did it feel to talk about this?
